# The remoulding of dietary effects on the fecundity / longevity trade-off in a social insect

**DOI:** 10.1186/s12864-023-09335-z

**Published:** 2023-05-05

**Authors:** Veronika Rau, Thomas Flatt, Judith Korb

**Affiliations:** 1 Evolutionary Biology & Ecology, University of Freiburg, Hauptstrasse 1, 79104 Freiburg (Brsg.), Germany; 2 Department of Biology, University of Fribourg, Chemin du Musée 10, CH-1700 Fribourg, Switzerland; 3 RIEL, Charles Darwin University Casuarina Campus, Ellengowan Drive, Darwin, NT0811 Australia

**Keywords:** Termites, Life-history, Protein-enrichment, Insulin-signalling, Juvenile hormone, Fecundity

## Abstract

**Background:**

In many organisms increased reproductive effort is associated with a shortened life span. This trade-off is reflected in conserved molecular pathways that link nutrient-sensing with fecundity and longevity. Social insect queens apparently defy the fecundity / longevity trade-off as they are both, extremely long-lived and highly fecund. Here, we have examined the effects of a protein-enriched diet on these life-history traits and on tissue-specific gene expression in a termite species of low social complexity.

**Results:**

On a colony level, we did not observe reduced lifespan and increased fecundity, effects typically seen in solitary model organisms, after protein enrichment. Instead, on the individual level mortality was reduced in queens that consumed more of the protein-enriched diet – and partially also in workers – while fecundity seemed unaffected. Our transcriptome analyses supported our life-history results. Consistent with life span extension, the expression of IIS (insulin/insulin-like growth factor 1 signalling) components was reduced in fat bodies after protein enrichment. Interestingly, however, genes involved in reproductive physiology (e.g., vitellogenin) were largely unaffected in fat body and head transcriptomes.

**Conclusion:**

These results suggest that IIS is decoupled from downstream fecundity-associated pathways, which can contribute to the remoulding of the fecundity/longevity trade-off in termites as compared to solitary insects.

**Supplementary Information:**

The online version contains supplementary material available at 10.1186/s12864-023-09335-z.

## Background

Diet affects life-history trade-offs, including fecundity and longevity, in solitary model organisms from nematode worms and fruit flies to mice and humans (e.g., [1–8]; reviews: [9–11] and references therein). Mechanistically, such trade-offs are thought to be regulated, at least in part, by two closely interacting pathways, the IIS (insulin/insulin-like growth factor 1 signalling) and TOR (target of rapamycin) pathways, which sense the availability of carbohydrates and amino acids, respectively. These pathways regulate downstream hormones (e.g., juvenile hormone, JH, a major gonadotropin in insects) and physiological processes related to life-history and somatic maintenance, such as fecundity or immune defence (e.g., [12, 13]). To emphasize the intricate interactions between these pathways and their effects on life history, this network has recently been dubbed the “TI-J-LiFe” network (short for TOR/IIS – JH – Lifespan/Fecundity). It is thought to be of key importance for our understanding of ageing, fecundity, and the fecundity/longevity trade-off in social insects [14].

Dietary restriction (DR; i.e. reduced food intake without malnutrition) can increase life span in numerous animal species (e.g., [2, 11]). More specifically, a low ratio of proteins relative to carbohydrates diet extends life span ([15]; reviews: [9–11] and references therein). Proteins (and their constituent amino acids) have life shortening effects (e.g., [16, 17]; see [18] for a review), while they are essential for reproduction. Accordingly, this protein effect has been associated with the common life-history trade-off between fecundity and longevity (though conflicting evidence exists, e.g. [9]).

The queens of eusocial insects (termites and social Hymenoptera such as ants and some bees and wasps) seem to have overcome the trade-off between fecundity and longevity. They (and in termites also kings) are typically the only individuals reproducing within a colony. Nevertheless, they live much longer than their non-reproducing worker nestmates [19, 20], with mating even leading to increased longevity (e.g., [21]), in contrast to other organisms (e.g., *Drosophila*) in which mating decreases life span (e.g., [22]). Indeed, social insect queens can live for decades and, in some species, can lay thousands of eggs per day. This strongly contrasts with the sterile workers that often live only a few months [20, 23], or with other insects such as *D. melanogaster* females, which can lay up to approximately 100 eggs per day and typically have an adult life span of approximately 30–40 days [9]. It is thus an interesting and largely open question how different diets affect life span and fecundity in social insects.

Similar to solitary animals, studies in social Hymenoptera have shown that workers fed on a high protein diet tend to die faster compared to a more carbohydrate-enriched diet (e.g. ants [24–26], bees: [27]). Much less is known for termites, which are social cockroaches (a monophyletic clade nested within the Blattodea) that evolved eusociality independent from social Hymenoptera (e.g., [28]). In the higher termite *Nasutitermes exitiosus* worker life span decreased with increasing protein collection [29], while the nitrogen-enrichment of wood blocks, in which termites were kept, increased the fecundity of neotenic replacement queens of the dampwood termite *Zootermopsis angusticollis* [30]. The studies so far mostly concentrated on either fecundity or survival, and there are no molecular studies in termites addressing this question.

**Fig. 1:**
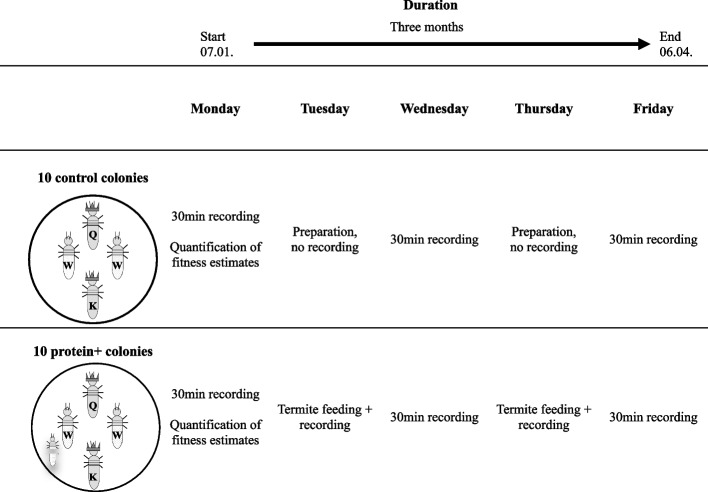
The weekly experimental procedure. Every Monday we noted the colony composition. Three times per week all colonies were recorded for 30 min. Twice per week, the treatment colonies (lower row) were fed with a dead termite (blurred individual) and the consumption was recorded. During that time the control colonies (top row) were prepared for recording but not actually filmed. The whole experiment was conducted over three months

Here, we tested the hypothesis that a protein-enriched diet influences the fecundity and longevity of the wood-dwelling (one-piece nester sensu [31]) termite species *Cryptotermes secundus* (Kalotermitidae). Wood-dwelling termites nest inside a piece of wood that serves as food and shelter and which the workers never leave to forage outside. As is typical for wood-dwelling species, *C. secundus* has a low social complexity with a few hundred workers that are totipotent immatures from which all reproductives (queens and kings) develop [32, 33]. Reproductives feed independently on the wood [34]. As the minimal amount of protein in wood-dwelling termites that do not forage for food (in contrast to the study on *N. exitiosus* by [29]) is fixed by the wood block the colony nests in [35], it is not possible to perform a DR study that reduces protein relative to carbohydrates in this species. Instead, we performed the opposite experiment, i.e., increasing the protein to carbohydrate ratio in their diet (hereafter, protein +), and comparing this to similarly handled but not protein-enriched control colonies (hereafter, ‘control’) (see Fig. 1). To provide a protein-enriched diet we used freshly killed termites; dead termites have an increased protein to carbohydrate ratio compared to the wood that is poor in nitrogen/protein (< 0.5% nitrogen) [36, 37]. Termite workers consist of about 20–30% crude protein and 1–3% crude fat (e.g. [38]). We chose this approach because enriching wood blocks with additional nitrogen often leads to developmental artefacts in *C. secundus* (intercastes; Korb, unpublished data), and termites would have no food choice. As is typical for termites, dead or injured nestmates are instantaneously consumed by all castes when they occur under natural conditions regardless if the colony is starving or not [37, 39]. This is thought to be an adaptation to recycling precious nitrogen in termites that feed on nitrogen-poor plant material [37]. It poses little risk of pathogen/immune infection as the pathogen load is low in drywood termites [40]. A further advantage of using corpses is that their consumption/uptake can be quantified, at least on a relative scale.

We aimed to manipulate protein availability relative to carbohydrates over a medium-term period analysing several fitness proxies and protein consumption (Fig. 1). Specifically, we tested whether a diet with an increased protein to carbohydrate ratio increases the fecundity of queens at the expense of reduced life span for queens and / or workers. To test for an effect of genetic background to diet manipulation, we used experimental colonies that were set up from larger stock colonies so that we obtained experimental colonies with the same and different genetic background. An earlier study in *C. secundus* has shown that the genetic background can influence a colonies/individuals response to a stimulus [41]. Contrary to that, stock colony (i.e., genetic background) had no significant effect on any of the fitness estimates in the current study, except for reproductive survival among the protein + colonies (see Supplementary Information: feeding results; Supplementary Information: Figure S1b). Additionally, we generated transcriptomes for heads with prothorax as well as fat bodies to analyse gene expression differences between control queens and those that received a protein-enriched diet. This allowed us to examine whether there are signs of ageing or fecundity effects that we might have missed at the phenotypic level. Furthermore, the transcriptomic analysis enabled us to obtain insights into the molecular underpinnings of fecundity and longevity in response to dietary manipulation. Comparing diet-induced gene expression changes, especially in the context of the TI-J-LiFe network, between the social termite and those known for solitary model organisms such as *Drosophila* contributes to our understanding of the remoulding of the fecundity /longevity trade-off with sociality.

## Results

### Components of fitness

The survival of the original queens (i.e., the queen present at the start of the experiment) did not differ between control and protein + colonies (Binomial GLMM: treatment: *N* = 19, *χ*^*2*^ = 1.39, *p* = 0.239) (Supplementary Information: Figure S1a). Similarly, no significant effect of treatment was found when analysing queen and king survival combined (see Supplementary Information, fitness results). One replacement queen developed in the control colonies and two replacement queens developed in the protein + queens because the original queens died.

**Fig. 2:**
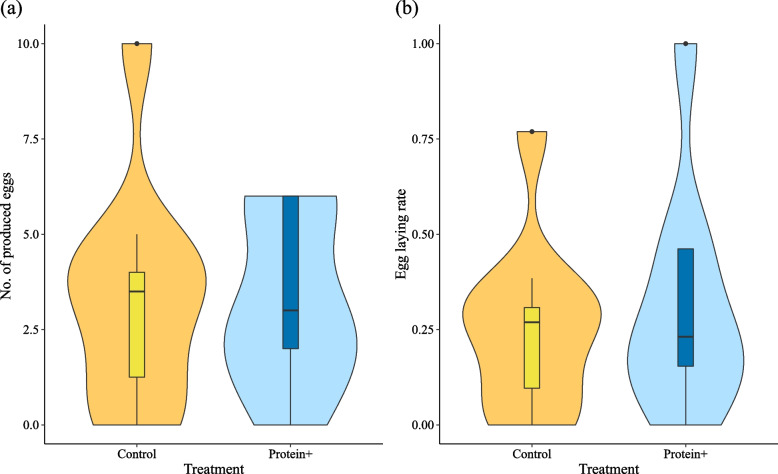
Queen fecundity. The number of eggs produced within a colony during the whole experimental period shown with (**a**) violin plots indicating the data distribution and boxplots indicating median (black bar), interquartile range (box) and total data range (whiskers and dots). The violin plots in (**b**) show the egg laying rate of original queens standardised by the time a queen was present. Control: yellow, protein + : blue. Treatment had neither a significant effect on (**a**) the total number of eggs produced nor on (**b**) the individual egg laying rate of original queens

In terms of queen fecundity, the total number of eggs produced within a colony did not differ between control and protein + colonies (Poisson GLMM: *N* = 20, *χ*^*2*^ = 0.54, *p* = 0.463) (Fig. 2a). The same was true for egg laying rate of original queens (Gaussian LMM: *N* = 19, *χ*^*2*^ = 0.59, *p* = 0.443). The analysis with original and replacement queens combined revealed qualitatively identical results (see Supplementary Information: fitness results; Supplementary Information: Figure S2). The egg production rate might seem low in this experiment (Fig. 2). Yet it is typical for young *C. secundus* queens. They lay only around a 20 eggs/year in their first year, under natural conditions [20] and in untreated laboratory colonies [41].

**Fig. 3:**
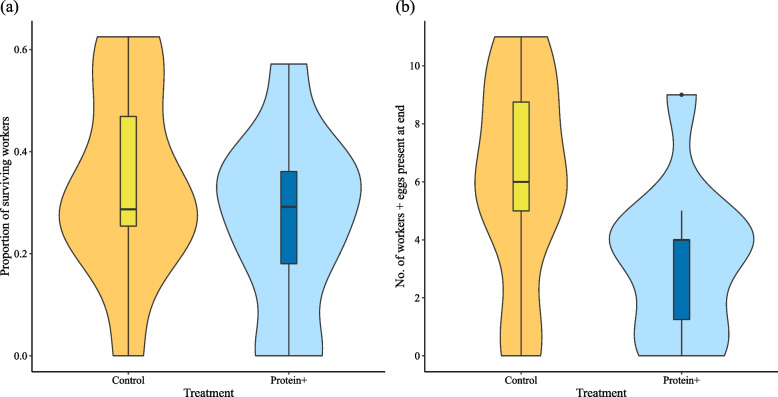
Survival of workers and total fitness estimates. The data are pooled by treatment with violin plots. Control: yellow, protein + : blue. Treatment had no significant effect on (**a**) the proportion of surviving workers nor on (**b**) the total fitness represented by the number of workers and eggs present at the end of the experiment

For the fitness components of the workers, the proportion of surviving workers did not differ between control and protein + treatment (Gaussian LMM: treatment: *N* = 20, *χ*^*2*^ = 0.94, *p* = 0.332) (Fig. 3a).

Total colony fitness also did not differ between control and protein + treatment (Poisson GLMM: *N* = 20, *χ*^*2*^ = 6.00e-04, *p* = 0.980) (Fig. 3b).

The number of workers present at the start positively affected the number of eggs produced within a colony and the total colony fitness (see Supplementary Information: fitness results; Supplementary Information: Figure S3, Figure S4).

Overall, the analyses of the fitness measures revealed no significant effects of treatment on worker and queen survival or on queen fecundity on the colony level.

### Feeding analysis

The analysis of all castes combined showed a strong influence of caste on survival (see Supplementary Information: feeding results) which potentially overshadowed the treatment effects.

**Fig. 4:**
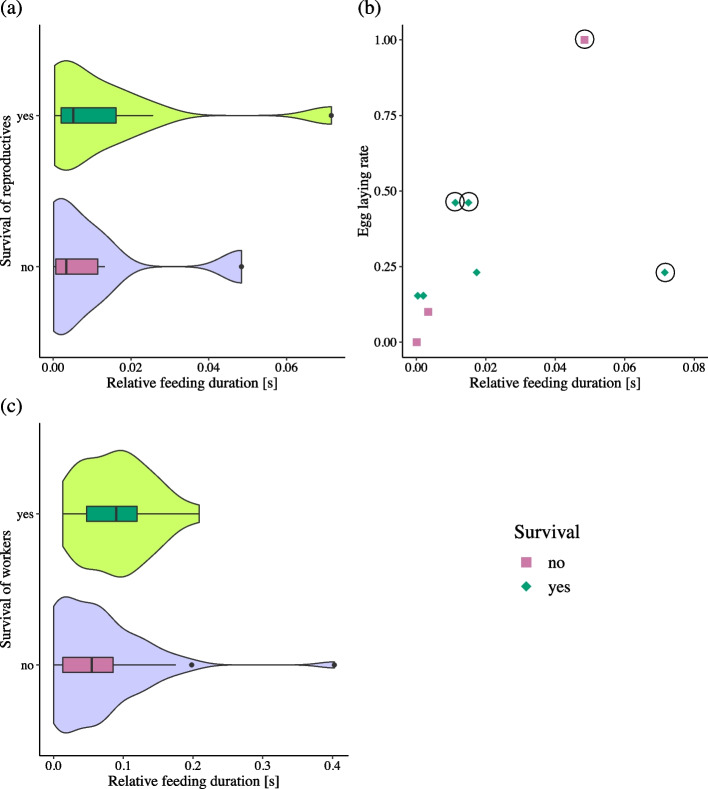
Feeding analysis. Shown are the relationships between relative feeding duration [s] and (**a**) reproductive survival, (**b**) queen fecundity (egg laying rate), and (**c**) worker survival. The relative feeding durations in (**a**) reproductives and (**c**) workers are shown with horizontal violin plots indicating the data distribution and boxplots indicating median (black bar), interquartile range (box) and total data range (whiskers and dots). The relationship between (**b**) queens’ feeding duration and egg laying rate is shown with dot plots including jitter to avoid overlapping of data points. Data from surviving individuals are shown in green (diamond shape), data from dead individuals in purple (squares). Samples used in the RNA-Seq analysis are marked with a black circle. Relative feeding duration had a significantly positive effect on (**a**) reproductive survival while (**b**) individual egg laying rate was not significantly influenced, and (**c**) workers’ survival was affected by trend by feeding duration

The caste-specific survival analyses revealed that reproductives which fed longer on the carcass were more likely to survive the experimental period (Binomial GLMM: *N* = 18, *χ*^*2*^ = 4.74, *p* = 0.029) (Fig. 4a). However, the egg laying rate of a queen was not significantly affected by relative feeding duration (Gaussian LMM: *N* = 9, *χ*^*2*^ = 1.37, *p* = 0.242) (Fig. 4b). Correlating queen survival with the mean relative feeding durations of all workers of a colony showed no significant association (Binomial GLM: queen survival: *N* = 9, *χ*^*2*^ = 10.90, *p* = 0.454). The same was true for queen feeding duration, it was not significantly influenced by mean worker feeding duration (Pearson’s correlation: queen relative feeding duration: *N* = 9, *r* = -0.17, *p* = 0.653).

For workers, we did not detect a significant effect of relative feeding duration on survival even though a tendency for a positive effect could be observed (Binomial GLMM: *N* = 81, *χ*^*2*^ = 2.84, *p* = 0.092 (Fig. 4c). Workers closer to death fed significantly shorter on the carcass (LMM: *N* = 51, *χ*^*2*^ = 10.24, *p* = 0.001), and there was also a significant effect of individual ID (LMM: *N* = 51, *χ*^*2*^ = 21.15, *p* = 4.242e-06). Reproductives showed no such patterns of feeding behaviour over time until death (LMM: day: *N* = 7, *χ*^*2*^ = 0.00, *p* = 1.000, individual ID: *N* = 7, *χ*^*2*^ = 0.30, *p* = 0.586).

### Differentially expressed genes (DEGs)

By chance, we obtained fat body transcriptomes mainly for queens that had long feeding durations on the carcass (see Fig. 4b).

For the head transcriptomes, we found only four genes that were significantly differentially expressed between protein + and control queens, with three DEGs being more highly expressed in the treatment group and one in the control group (Supplementary Information: Table S1). There was some evidence for differential expression of genes associated with IIS, TOR and JH/fecundity (TI-J-LiFe network) upon protein + treatment in the head (Supplementary Information: Figure S7). In contrast, 1035 genes were significantly differentially expressed in fat bodies. Four hundred twenty-one genes were more highly expressed in the protein + group and 615 were more highly expressed in the control group (Supplementary Information: Table S1).

For both tissues, we had data from original and replacement queens. These two groups did not obviously differ in their expression profile as revealed by PCA analyses (Supplementary Information: Figure S6). So, we treated them as one group.

#### Genes more highly expressed under protein + conditions in fat bodies

**Fig. 5:**
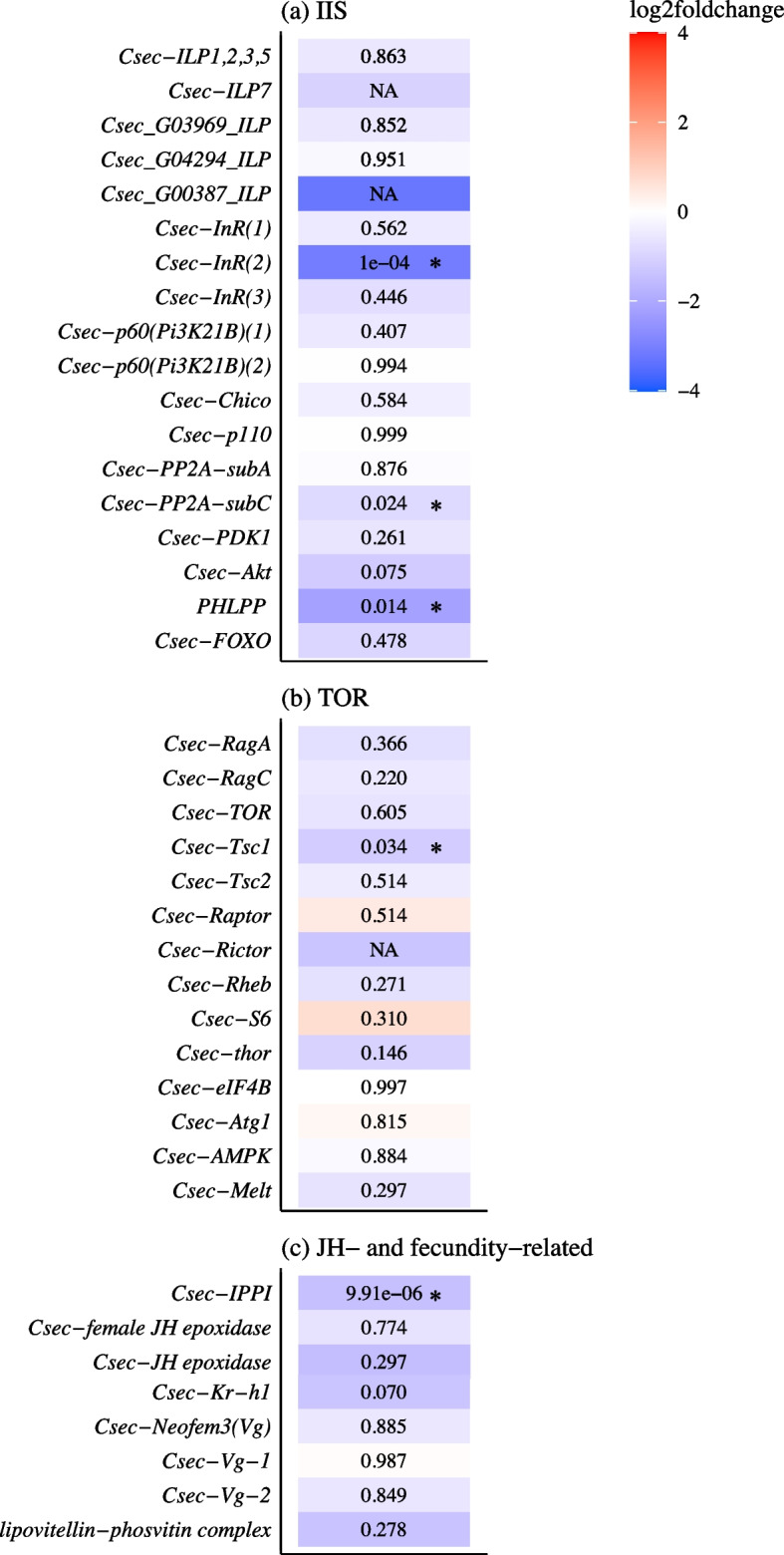
Heatmap for differential gene expression analysis in fat bodies. Shown are the results of the differential gene expression analysis in fat bodies for genes related to IIS/TOR and genes indicating high JH titers and fecundity in *C. secundus*. The colour symbolizes the log2foldchange value with red indicating an upregulation and blue a downregulation of the respective gene under protein + conditions. The adjusted *p*-values are given in the tiles, significant DEGs are marked with a star

We observed no particular pattern among the genes upregulated under protein + conditions (Supplementary Information: Table S1). In contrast to expectation, neither *Csec-TOR* nor any gene from the TOR pathway were significantly more highly expressed in the protein + group as compared to control, although we had manipulated protein availability which is normally sensed by this pathway (Fig. 5, Supplementary Information: Table S2). We also did not detect upregulation of genes related to fecundity as might have been expected, given that protein-enhanced diets typically increase fecundity. None of the three reproductive/queen associated termite vitellogenins (Vgs) were among the protein + DEGs nor were JH epoxidases, which are associated with high JH biosynthesis in *C. secundus* [42] (Fig. 5).

Additionally, an oxysterol binding protein (*OSBP*) and a putative sterol dehydrogenase were among the DEGs more highly expressed in protein + queens.

We only found a minor overlap (30 of 421 genes) between DEGs that were more highly expressed in protein + queens and old-age-genes (i.e., genes characteristic for old *C. secundus* queens) (1056 genes). The number of overlapping genes was not different from chance expectation when randomly drawing genes (contingency analysis: *χ*^*2*^ = 0.91, *df* = 1, *p* = 0.341). This is consistent with the fitness data above, suggesting that a protein-enriched diet does not lead to increased ageing.

#### Genes more weakly expressed under protein + conditions

For genes downregulated under protein + conditions, we found some striking patterns. As expected for a shift towards a less carbohydrate-biased diet, several genes from the IIS pathways were among the DEGs more weakly expressed in the protein + group (Fig. 5). While the five identified insulin-like peptides (*ILPs*) were not affected, the expression of *Csec-InR(2)*, a supposed ‘entry-point’ of the IIS pathway was influenced. Additionally, *Csec-Akt*, the ortholog of *D. melanogaster Akt* /PKB (protein kinase B) [42], was not significantly affected but a trend was observable (Fig. 5). The latter is the major ‘exit point’ of the IIS that controls many downstream processes related, for instance, to ageing and fecundity. *Csec-phlpp*, a homologue of a gene which deactivates Akt by dephosphorylation in humans as well as *D. melanogaster* [43], was also downregulated under protein + conditions, which might imply that negative feedback regulation of Akt is not active, if Akt is low. Akt also provides a link between the IIS and the TOR pathway by inhibiting the TSC complex (Tsc1/Tsc2), which further inhibits TOR [44]. However, *Csec-tsc1* was also among the DEGs significantly downregulated under protein + conditions (Fig. 5). Among the potentially fecundity-related genes, isopentenyl-diphosphate delta-isomerase, which is supposedly involved in JH biosynthesis, was significantly downregulated under protein + conditions (Fig. 5). *Csec-Kr-h1*, an ortholog of *Kr-h1* (Kruppel homolog 1), the early JH response gene in the cockroach *Blattella germanica* [45] and *D. melanogaster* [46], might be down-regulated by protein enrichment as it affected by trend (Fig. 5). This could potentially imply reduced JH signalling and hence reduced fecundity. However, none of the other genes associated with JH biosynthesis was affected (Supplementary Information: Table S2), including *Csec-female-JH epoxidase* (Fig. 5), which is supposed to catalyse the last step in JH production in *C. secundus* queens/females [42]. Also, the expression of three termite Vg-genes, which are associated with reproduction in termites [42], was unaffected (Fig. 5).

We also found two genes annotated as fatty acid synthases 1 (*FASN1*), *Csec-AC011*, *lip3_5* and SREBP cleavage activating protein indicating that lipid metabolism might be affected by the protein- (and slightly fat-) enriched diet. In addition, a gene annotated as *Pepck1* was among the DEGs, which is supposed to be involved in glucogenesis.

Again, we only found a small overlap (36 of 615 genes) between the DEGs more weakly expressed under protein + conditions and old-age-genes (1056 genes). This number did not differ from random expectation (contingency analysis: *χ*^*2*^ = 1.19e-29, *df* = 1, *p* = 1.000).

#### GO term analysis

The GO term enrichment analysis revealed nine significantly overrepresented biological processes for fat bodies under protein + conditions, among them “galactose metabolic process” (GO:0006012), nine cellular components (e.g., “ribosome”; GO: 0005840) and 18 significantly overrepresented molecular functions such as “lipid binding” (GO:0008289) (Supplementary Information: Table S3).

In the fat bodies of the control group, 31 biological processes were significantly overrepresented including “glucogenesis” (GO:0006094), “carbohydrate metabolic process” (GO:0005975) and “glycoprotein catabolic process” (GO:0006516) (Supplementary Information: Table S3), showing a metabolic signal. Three cellular components were overrepresented and 45 molecular functions including “triglyceride lipase activity” (GO:0004806) (Supplementary Information: Table S3).

## Discussion

Here we have studied the effects of a mainly protein-enriched diet on fecundity and longevity in a wood-dwelling termite by providing termite corpses with a higher protein to carbohydrate content than their standard food, wood (protein content of termite workers about 20–30% compared to less than 0.5% in wood; [36–38]). Interestingly, in marked contrast to expectation, we did not find any evidence for increased fecundity and reduced longevity, as commonly observed in solitary insects such as *D. melanogaster*. Both our analyses of fitness components and of the gene expression data suggest that queen fecundity was unaffected by dietary manipulation. By contrast, the longevity of queens – and maybe also that of workers – increased on the individual level as individuals spending longer durations on feeding corpses were more likely to survive (Figs. 2 and 4). This seems also to be supported at the gene expression level, which revealed a signal of reduced IIS signalling in fat bodies after protein enrichment.

### Fitness effects of a protein-enriched diet

Surprisingly, we did not find an effect of our diet manipulation on fitness measures at the colony level when comparing protein + with control colonies (Figs. 2 and 3). However, we detected clear signals at the individual level within the protein + colonies: a longer feeding duration on the additional protein source, the carcass, was associated with increased survival for reproductives and, with weaker evidence, workers, while fecundity seems unaffected (Fig. 4). This suggests that the lack of an obvious effect at the colony level is not due to the lack of a treatment effect. Rather inter-individual differences in feeding at the carcass (Fig. 4) might have led to an overall non-significant effect at the colony level. These results highlight the importance of measuring/estimating food intake rates at the individual level in such experiments.

The causes of the observed intraspecific variation remain unclear. The genetic background did not seem to play an important role (except maybe one stock colony, no. 5, but experimental colonies from this stock colony had high mortality rates regardless of treatment, Figure S1b). This contrasts with a previous study, in which effects of temperature variability on fitness proxies was tested, and in which the genetic background influenced the response of a colony [41]. Stress associated with the death and replacement of a queen might have contributed to inter-colonial variation. However, this is difficult to test as we had only three replacements, two protein + and one control colony. Other fitness measures as well as the gene expression data do not indicate any obvious effect of queen replacement (e.g., survival of workers, total fitness estimates, Figure S4; gene expression, Figure S6).

We also did not detect a social effect of nestmate workers on reproductive survival as neither queen survival nor queen feeding duration was correlated with the feeding time of workers or worker survival. We cannot completely rule out that the effect observed at the individual level is causally reversed, i.e., that those individuals which were less likely to die were those that feed more. Yet, we think this is unlikely as we tried to account for this effect by calculating and using the relative feeding time, i.e., feeding time corrected for observation period when the animal was present. Second, workers closer to death did show less feeding. However, this effect was not visible in reproductives in which increased survival correlated with extended feeding (see results), suggesting that an individual’s health did not influence an individual’s feeding duration. Thus, we conclude that increased feeding at the carcass – and hence a probably higher protein intake – resulted in increased survival of reproductives. Other nutrimental components, like a slightly increased fat content of about 1–3% in termite workers [38], might have contributed to this effect. Yet we think that the major effect is due to the strong protein enrichment (20–30%) as is also indicated by the gene expression results (see below).

Our results differ when compared to other studies. In solitary insects like *D. melanogaster* as well as social Hymenoptera like ants and the honeybee, a protein-enhanced diet generally leads to increased fecundity at the expense of longevity (*D. melanogaster*: e.g. [15]; ants: [24–26]; honeybee: [27]).

Other termite studies that tested protein or nitrogen availability are scarce and none investigated effects on survival as well as fecundity. Nitrogen-enrichment of the wood in the wood-dwelling termite *Z. angusticollis* increased the fecundity of young replacement reproductives, though not founding queens [30]. As complete wood blocks were enriched with nitrogen in the *Z. angusticollis* study, the termites could not adjust nitrogen intake separately from wood intake, which they could do in our study. The foraging workers of the termite species *N. exitiosus* did not regulate the quantity of food they collected and only avoided diets very high in protein which have a negative effect on worker life span [29, 47]. Direct comparisons to our study are difficult as treatments differed and both of these studies did not investigate life history consequences at the individual scale, i.e., relating individual consumption rates to individual life span/fecundity.

### Mechanisms associated with a protein-enriched diet

#### Tissue- and nutrient effects

Our transcriptome analyses revealed only four DEGs for the head/prothorax samples while we found over 1000 genes to be differentially expressed in the fat body of queens when we manipulated diet. This is in line with the vital part of fat bodies in insect metabolism [48–50]. A DR study in *D. melanogaster* revealed very tissue-specific effects with fat bodies to be more affected than other body parts (e.g. brain) [51]. Our gene expression study also revealed a clear metabolic signal of diet manipulation in the fat bodies. This included carbohydrate-related GO terms under control conditions indicating that carbohydrates are of less importance in the queens provided with additional protein from the dead termites (Supplementary Information: Table S3). In addition, we found a GO signal for triglyceride lipase activity overrepresented in control queens that may indicate increased lipid mobilisation. This might reflect the increased availability of fat for queens provided with dead termites which consisted of about 1–3% crude fat [38]. Yet, how such a lipid signal should affect life histories is not clear. The effect on lifespan varies between different genes. A knockdown of the triglyceride lipase *brummer* leads to a shortened lifespan in *D. melanogaster* [52] while *lipase 3* is upregulated in starved and aged flies and has been associated with a shortened lifespan [53]. Regarding fecundity, there are studies linking triglyceride lipase activity positively to fecundity in *Drosophila* [54–56].

A recent study in *D. melanogaster* highlighted the importance of dietary sterol for fecundity, especially under varying protein to carbohydrate ratios [57]. The positive effect of protein on fecundity is compromised if sterol is absent from the diet whereas supplementary sterol can dilute the negative effect of protein on lifespan [57]. However, only very few genes related to sterol were differentially expressed although the queens ingested fat together with protein. As we found no fecundity effect neither in the fitness analysis nor on a genetic level, we assume that the intake of sterol had no observable effect on fecundity. This is in line with the equally absent effect of protein on fecundity. It is possible that the observed neutral or even positive effect of protein on lifespan was mediated by the accompanied provision of dietary sterol, though this seems unlikely considering the weak genetic signal of sterol.

Our transcriptome data revealed an effect of diet manipulation at the colony level but only at the individual and not at the colony level for the fitness data. This discrepancy might be explained by the fact that, due to chance, we mainly had fat body transcriptome data for those individuals that had fed longer on the carcass and which therefore are assumed to have ingested on average more protein (see Fig. 4b). This increased the signal to noise ratio in the RNA-Seq dataset compared to the fitness dataset because the less feeding (and hence, more control-like individuals) individuals were absent in the protein + gene expression dataset which increased the protein + signal. Hence, we see the survival effects more clearly in the gene expression data.

#### IIS, TOR, and longevity

**Fig. 6:**
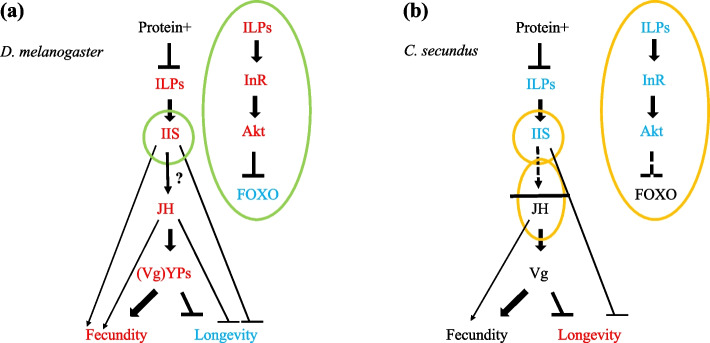
Models of the IIS-JH-Vg/YPs circuit showing the effect of a protein-enriched diet (without overfeeding) in (**a**) the solitary insect, *D. melanogaster*, and in (**b**) the termite *C. secundus* (current study), with key genes of the IIS pathway (including FOXO) depicted in the green and orange circles. Arrow: activation; stop bar: repression; dashed lines: potential re-wiring points; ?: connecting links unclear; red: up-regulation, blue: down-regulation, black: unaffected. In contrast to *D. melanogaster*, increased protein levels seem to lead to a downregulation of IIS in *C. secundus*, without effects on FOXO expression. Similarly, changes in IIS activity in the termite queen did not (strongly) impact JH biosynthesis (as indicated by the horizontal bar) unlike in *D. melanogaster*. Thus, fecundity is unaffected by protein enrichment in the termite, while it generally increases fecundity in solitary insects such as *D. melanogaster*. The negative linkage between IIS and longevity seems conserved as is the positive association between JH and fecundity, as reflected in the regulation of Vg and YP (the functional fly ‘equivalent’ of Vg) by JH. Figure (**a**) adapted from [61]

Like the GO terms, analyses of nutrient-related pathways also reflected a clear shift to a less carbohydrate rich diet under protein + conditions. Several important genes (incl. *Csec-InRs* and *Csec-Akt*) of the carbohydrate sensing IIS pathway were downregulated in fat bodies under protein + compared to control conditions (Fig. 5, Fig. 6). A down-regulation of the IIS pathway is in line with increased survival of queens under protein + conditions as lower IIS signalling is generally associated with increased life span (e.g., *D. melanogaster*: [58, 59]). However, down-regulation of IIS in *D. melanogaster* is untypical for a protein-enriched diet (reviews: [9–11] and references therein) (Fig. 6). A similar effect of decreased IIS activity as in *C. secundus* was only observed in *D. melanogaster* after potential *overfeeding* with yeast (protein) [60].

Overall, the amino-acid sensing TOR pathway seemed less affected, though a downregulation of the potential TOR inhibitor *Csec-Tsc1* indicates crosstalk between IIS and TOR, which however did not lead to an upregulation of TOR (Fig. 5). The missing effect on the TOR pathway was surprising as TOR senses amino acids but it is in line with the lack of a fecundity effect at the phenotypic level.

Strikingly, the expression of *Csec-FOXO* is unaffected by the protein addition (Figs. 5 and 6). FOXO is a regulator of many downstream processes prolonging life span (e.g., [62–66]). The activity of this transcription factor is generally inhibited by Akt, which prevents the translocation of FOXO from the cytoplasm to the nucleus via phosphorylation [66, 67]. The unchanged expression of the anti-ageing gene *Csec-FOXO* together with its implied lowered inhibition by Akt is in line with the fitness data of increased survival of reproductives that fed longer on the carcass. As we included, by chance, mainly these longer-feeding queens in our transcriptome analysis (see also Fig. 4b), we are able to see an effect on genes related to survival although we do not see this effect in the fitness measurement at the colony level. The old-age-gene analyses which revealed no signs of an enrichment of ageing genes under protein + conditions further supported our results.

#### Fecundity associated processes

Supporting the life history measures (Fig. 2), we did not detect any effect of protein-enrichment on fecundity-related genes. None of the DEGs were associated with fecundity and the three annotated termite Vgs, which characterise reproductives [42, 68], were not affected at all (Fig. 5, *p* > 0.800). This result is striking given we analysed transcriptomes of fat bodies, the tissue where most Vg production occurs [48].

Our results contrast fundamentally with those for solitary insects, in which a protein-enriched diet leads to increased fecundity (e.g., [15, 60, 69]). A positive association between IIS and JH (e.g. *B. germanica*: [70], *D. melanogaster*: [71]) results in enhanced fecundity but shortened life span (e.g., [72] and references therein) (Fig. 6). In *C. secundus*, as in other termites, queen fecundity is also JH dependent and high JH titres are associated with an upregulation of the three termite Vgs [42, 73, 74]. From former studies in termites [30], we expected young neotenic queens to be mostly affected by the protein availability. However, protein treatment, and the associated IIS downregulation, had no strong effect at the JH level in our study (Fig. 6). None of the key JH biosynthesis genes (e.g., JH epoxidases, which supposedly catalyse the last step in JH production) or JH signalling genes were affected, except for marginal significant downregulation of *Csec-Krh1* under protein + conditions (Fig. 5, Supplementary Information: Table S2). The lack of an effect on JH biosynthesis genes could be explained by the fact that we refer to fat body results here, while JH biosynthesis occurs in the corpora allata of the head/prothorax. However, we also did not find an effect of protein-enrichment in our head/prothorax transcriptomes (see results; Supplementary Information: Table S1, Table S2). The latter is unlikely to be an artefact as we detected JH-fecundity signals in head/prothorax tissues of *C. secundus* in comparable, former transcriptome studies [42], demonstrating that it is possible to detect JH signals in head transcriptomes.

Overall, our results consistently suggest that – like the fitness data implied – fecundity was not affected by providing corpses of termites, a diet that is especially rich in protein, compared to their standard-diet of wood [36–38]. This differs fundamentally from other insect studies, in which dietary protein content is manipulated (e.g., [15, 24, 26, 27, 57, 60]) and in which a link between longevity/survival and fecundity exists that is mediated via the IIS and TOR pathway. Thus, we propose that an uncoupling occurs between IIS-associated lifespan regulation and JH-associated fecundity which may underlie the overcoming of the fecundity / longevity trade-off in termite queens (Fig. 6). This hypothesis can be tested in upcoming studies.

## Conclusion

Our study suggests that a protein-enriched diet does not increase the fecundity of the wood-dwelling termite *C. secundus*, neither at the colony nor the individual level, as shown by our fitness and RNA-Seq analyses. In addition, survival was not negatively affected, as opposed to what has been previously found in other (social) insect species. At the individual level of queens, we even found indications for a positive effect of a protein-enriched diet as we detected signs of lower IIS activity which is usually associated with increased lifespan. In conclusion, we suggest that an uncoupling might have occurred within the IIS-JH-Vg axis, thereby allowing lifespan and fecundity to be regulated separately. This might explain the remoulded fecundity/ longevity relationship in termite queens.

## Material and methods

### Colony collection, maintenance, and experimental design

The *C. secundus* colonies used in our study were derived from six monogamous mature colonies collected in spring 2019 from mangroves near Palmerston-Channel Island, Northern Territory, Australia (12°50’S 131°00’E). Using these stock colonies, 24 experimental colonies were directly set up in Australia by placing 30 workers from a stock colony in *Pinus radiata* wood blocks providing abundant food conditions [32, 33]. Among these workers, a pair of neotenic reproductives (hereafter, reproductives, or king and queen) develops which starts to lay eggs. Thus, we obtain functional mature colonies with young fertile reproductives of similar age and comparable colony sizes (reflecting young colonies). No soldiers were present as expected at this colony size [75]. By applying this technique, we could standardize colonies and reduce noise, e.g., from age differences of queens and large differences in colony size. In addition, we had neotenic queens that were supposed to be more sensitive to protein/nitrogen availability than primary queens as a former study implied [30]. The colonies were transported to Germany where they were kept in climate chambers with a temperature of 27 °C and 70% relative air humidity, the optimal maintenance conditions for *C. secundus* [32]. From the 24 colonies, only 20 developed into functional colonies with fertile reproductives; these were used for the experiment (hereafter, experimental colonies). The colonies were randomly allocated to the two treatments but ensuring that both treatment groups received colonies from each stock colony. The use of experimental colonies coming from the same stock colony allowed us to detect potential genetic background effects and to observe possible interactions between genetic background and treatment. Colony sizes at the start of the experiment did not differ significantly between colonies allocated to protein + versus control conditions (Mann Whitney-U Test: *N* = 20, *W* = 70, *p* = 0.139). Yet, as there might be subtle colony size effects, we included colony size as potential confounding factor in our fitness analyses (see below).

Out of the 20 experimental colonies, ten colonies were provided with a dead termite individual twice a week (Tuesday, Thursday) for three months (hereafter, for simplicity protein + condition; Fig. 1). This reflected a substantial increase in protein availability (total of 26 termites per colony) as dead termites, the major natural source of protein, occur rarely in colonies consisting of 10^th^ to a few hundred of individuals (estimation based on observations and worker mortality for undisturbed conditions: < 1 termite / months). The termites used for feeding were collected from other colonies of *C. secundus* and the closely related species *Cryptotermes domesticus*, which naturally co-nests in the same trees with *C. secundus*. Healthy termites without signs of infection were freshly killed before feeding and their abdomen was ripped open after placing them into the colony. The leaking haemolymph triggered the other termites to immediately consume the dead ‘nestmate’. The other ten colonies were handled in the same manner as the protein + colonies but without providing additional protein (hereafter, control condition; Fig. 1).

The whole experiment lasted for three months. All termites from a given colony were individually marked with enamel paint (Revell) to be able to track them throughout the experiment. Three times a week (Monday, Wednesday, Friday), all colonies were filmed for 30 min to observe the behaviour of individuals (Fig. 1). In addition, the protein + colonies were always filmed during the feeding process until only the hard head capsule and cuticle parts of the provided dead termite were left to quantify the amount of protein-enriched food each termite consumed. The ten control colonies were not filmed during that time but handled in the same way as the protein + colonies.

### Fitness components

We analysed the following proxies for survival: for reproductives, we used (i) a binary variable indicating whether the queen and king present at the start (original queen / original king) both survived the three months experimental period and (ii) the survival of the original queen alone as binary variable. For protein + we only had nine queens as we could not use one queen for technical reasons. To test for an effect of increased protein availability on queen fecundity, we quantified (i) the total number of eggs produced in a colony during the three months of the experiment, and (ii) the egg laying rate of each queen. For egg laying rate, we divided the number of eggs produced by a queen by the number of weeks the queen was present (maximum 13 weeks). We calculated the egg laying rate (i) for original queens only and (ii) for original and replacement queens (see Supplementary Information: Table S4). Measuring egg numbers directly was possible because *C. secundus* queens have low fecundity. Young queens, like the ones we used, produce only around 20 eggs per year.

The effect of protein + treatment on the workers was tested by calculating the proportion of surviving workers over the three months experimental period. By using the proportion rather than the absolute numbers, we accounted for slightly different colony sizes at the start of the experiment.

In addition, we compared total colony fitness between control and protein + colonies by using the number of alive workers and eggs present at the last day of the experiment. This is a suitable fitness proxy in *C. secundus* because workers are totipotent immatures from which the reproductives develop, as is typical for wood-dwelling termite species (i.e., no bifurcation during development into a line from which apterous workers *versus* winged sexuals develop [75, 76]). Under abundant food conditions, workers tend to stay in the nest while under scarce conditions they more likely develop into reproductives and disperse [77, 78].

No alates developed during the experimental period, as expected for the time of the year when the study was performed. Soldiers were not present in the colonies as is typical for *C. secundus* colonies with an age of less than one year.

To test the effect of protein availability on the diverse fitness proxies, we used generalised linear mixed models (GLMM) and generalised linear models (GLM) with the packages lme4 (version 1.1–26; [79]) and lmerTest (version 3.1–3; [80]) in *R* (version 4.0.3; [81]) with the appropriate family functions. For the GLMMs we used “treatment” as fixed factor, the number of workers at the start of the experiment (“start”) as a covariate and “stock colony” (indicating genetic background) as random factor. The fitness estimates were tested by removing a term and comparing the models with likelihood ratio tests. Based on the results of the Akaike Information Criterion (AIC, [82]) we did not include interaction between “treatment” and “start”. The test for the proportion of surviving workers was performed without the covariate “start”. Tests were two-tailed, and statistical significance was defined as *p* < 0.05, while a *p*-value between 0.05 and 0.1 was considered a trend. We used the false discovery rate (FDR) approach [83] to correct for multiple comparisons when appropriate. We always obtained qualitatively identical results when analysing the data using ‘simple’ statistics such as the corresponding Fisher’s exact tests or rank tests. Results were visualized using the package ggplot2 [84].

### Quantification of protein consumption

Since we could not measure the exact amount of protein consumed by a termite, we used the feeding time on the carcass as a proxy for protein consumption. As termite carcasses have a high protein content of around 20–30% [38], we assumed that an individual with a longer feeding duration on the carcass would also ingest on average more protein than an individual feeding on the carcass for only a fraction of the time. To this end, 99 individuals and more than 560 h of videos were analysed using the software BORIS [85]. The time a termite fed directly on the carcass was quantified for each feeding event. We thus obtained the total duration of how long a termite fed per feeding event as well as the duration over the complete experimental period of three months. Because some individuals died during the experimental period, we standardized these feeding times by dividing the total feeding time of an individual by the total time an individual was observed (hereafter, relative feeding duration) (Supplementary Information: Table S5). Using the total feeding time would bias our results since total feeding time is positively correlated with survival.

To test how relative feeding duration influenced worker and reproductive survival and queen fecundity, we used different approaches. All following analyses were exclusively performed on protein + colonies. First, we performed a GLMM with “survival” as the response variable, “relative feeding duration” as the covariate, and using “caste” as fixed and “stock colony” as random factors. Because caste had a highly significant influence on survival with workers feeding longer than queens, we additionally performed GLMMs and GLMs for each caste separately using likelihood ratio tests with “survival” as response variable, “relative feeding duration” as the covariate and “stock colony” as the random factor. To test the effect of feeding duration on the queen’s fecundity we used “individual egg laying rate” as the response variable, “relative feeding duration” as the covariate and “stock colony” as the random factor. The terms were removed one at a time and the models were then compared with likelihood ratio tests. In addition, we examined whether queen survival and queen relative feeding duration correlated with worker relative feeding duration (Supplementary Information: Table S5). To do so, we calculated the mean relative feeding duration of all workers per colony and performed logistic regressions with the appropriate family functions; “queen relative feeding duration” or “queen survival” were used as the response variables and “mean relative feeding duration of workers per colony” was used as the predictor.

In addition, we considered potential confounding factors which might have influenced feeding behaviour. To test if individuals closer to their death fed less on the carcass, we performed two linear mixed models (LMM) for dead workers and reproductives.; we used the “individual feeding duration per day” as the response variable, the “days” an individual was alive as the covariate and “individual number” (i.e., ID of an individual) as random factor. All tests were two-tailed, and significance was defined as *p* < 0.05 while *p* < 0.1 was defined as trend. Whenever appropriate, we used FDR [83] to correct for multiple comparisons. All models were performed in *R* with the packages lme4 (version 1.1–26; [79]) and lmerTest (version 3.1.3; [80]). Results were visualized using the package ggplot2 (version 3.3.5; [84]).

### Transcriptome preparation

All queens present at the end of the experiment were used to generate transcriptomes of heads (plus prothorax) and fat bodies, separately, without pooling samples (Supplementary Information: Table S6). Hence, ten samples were collected from the ten control colonies and seven samples were collected from the nine protein + colonies (two protein + colonies were dead at the end of the experiment). The protein + colonies were last provided with a dead termite five days before queen collection. Note, not all collected queens had been present at the start of the experiment (i.e., not all queens were original queens); one control queen and one protein + queen that died were replaced by new developing queens during the experiment. These two replacement queens did not obviously differ in their expression profile from that of the original queens (Supplementary Information: Figure S6).

Individuals were killed in a petri dish on ice, and head plus prothorax (hereafter ‘head’) was separated from the body and directly transferred to RNA later ® (Qiagen). The gut was removed and discarded, and fat bodies were collected from the abdomen by dissolving and absorbing them with 10 μl of PBS buffer. Both tissues were stored at -80 °C until isolation. The remains of thorax and abdomen (including e.g., ovaries) were transferred to RNA later and preserved at -80 °C. We performed the RNA extraction according to an in-house protocol with Trizol (see e.g., [41, 86]). We used the same protocol for both tissues but adjusted the volumes of the added chemicals: heads were isolated with 100% volume and fat bodies with 25% volume of the chemicals. RNA samples were sent on dry ice to BGI Tech Solutions Co. (Hong Kong); sequencing was performed at BGI Shenzhen (PR China). BGI prepared cDNA libraries using NEBNext Multiplex Oligos for Illumina (96 Unique Dual Index Primer Pairs) following their internal and proprietary standard operating procedure. Paired-end 150 bp sequencing was performed on an Illumina HiSeq X Ten platform, generating around 4GBases data per sample.

### RNA-seq data analysis

The transcriptome data were prepared for analysis as described in [41]. For fat bodies, some samples had to be excluded from the analysis due to low mapping rates, so we had four samples from the protein + condition and five control samples. By chance, the fat body samples consisted of queens that had longer feeding durations (marked in Fig. 4b). We performed gene expression analysis with tissue and treatment as two independent variables using the generalized negative binominal model implemented in DESeq2 (version 1.30.1; [87]), which internally normalizes the read counts (Supplementary Information: Table S7). The program calculates *p*-values using Wald statistics and corrects for multiple testing using the FDR approach [83]. We defined significance as* p* < 0.05 and a trend as *p* < 0.1. To test for potential signs of ageing, the differentially expressed genes (DEGs) between the treatments within a tissue were compared with genes characteristic for old queens (hereafter: old-age genes; [86]); the resulting overlaps were tested with *χ*^2^ tests to see if more genes overlapped than expected by chance. Ideally, we would have also analysed the transcriptome data set in relation to the feeding duration of the queens. However, this was not possible due to too low samples sizes for such analyses. We also performed a principal component analysis (PCA) implemented in DESeq2 with the normalized count data for both tissues combined and for heads and fat bodies separately (Supplementary Information: Figure S6). In addition, we used a GO term enrichment analysis for biological processes, molecular functions and cellular components implemented in the package topGO in *R* (version 2.42.0; [88]), with the default algorithm weight01, and Fisher’s exact tests.

### Annotation

Different approaches were used to annotate the DEGs. First, the existing annotation of the *C. secundus* genome [89] and the list of TI-J-LiFe genes (see e.g., [42]) were used. TI-J-LiFe genes are indicated by *Csec-genename* as they are manually curated genes with gene trees. For unannotated genes, we retrieved putative functions by performing a BLAST search against the fruit fly *D. melanogaster*, the fungus-growing termite *Macrotermes natalensis* and the dampwood termite *Zootermopsis nevadensis* using local blastp (version 2.9.0; [90]) with a threshold e-value of 1e-05. For *D. melanogaster*, we retrieved gene annotation from FlyBase [91], for *M*. *natalensis* from [92] and for *Z. nevadensis* from the nr database from NCBI [93]. GO terms were provided by a search against the Interpro Database (InterProScan-v5.46–81.0, [94].

## Supplementary Information

**Additional file 1.** Supplementary Information contains supplementary results of the fitness and feeding analysis, PCA, Heatmap for important TI-J-LiFe genes in heads.

**Additional file 2:** **Table S1.** Contains treatment-associated DEGs in heads and fat bodies.

**Additional file 3:** **Table S2.** Contains differential gene expression data of TI-J-LiFe genes in heads and fat bodies.

**Additional file 4:** **Table S3.** Contains GO terms of heads and fat bodies.

**Additional file 5:** **Table S4.** Contains fitness data of the 20 experimental colonies.

**Additional file 6:** **Table S5.** Contains feeding duration data of the 10 protein+ colonies.

**Additional file 7:** **Table S6.** Contains information about the 24 samples used for transcriptome generation and RNA-Seq data analysis.

**Additional file 8:** **Table S7.** Contains normalized counts of all expressed genes in this experiment.

## Data Availability

The datasets supporting the conclusions of this article are included within the article and its additional files except the raw sequencing reads that are available on NCBI (BioProject Accession Number: PRJNA746710).
