# Supplementary material for: The remoulding of dietary effects on the fecundity / longevity trade-off in a social insect
Source: BMC Genomics. 2023 May 5;24:244. doi: 10.1186/s12864-023-09335-z (PMC10163710; doi:10.1186/s12864-023-09335-z)
Supplement: Supplementary file 1 — Additional file 1. Supplementary Information contains supplementary results of the fitness and feeding analysis, PCA, Heatmap for important TI-J-LiFe genes in heads. [file 12864_2023_9335_MOESM1_ESM.docx]

**Supplementary Information**

Title: Re-moulding of dietary effects on the fecundity / longevity trade-off in a termite species

Veronika Rau^1^, Thomas Flatt^2^ & Judith Korb^1,3^

^1^ Evolutionary Biology & Ecology, University of Freiburg, Hauptstrasse 1, 79104 Freiburg (Brsg.), Germany

^2^ Department of Biology, University of Fribourg, Chemin du Musée 10, CH-1700 Fribourg, Switzerland

^3^ RIEL, Charles Darwin University Casuarina Campus, Ellengowan Drive, Darwin, NT0811, Australia

**Supplementary Results**

**Fitness results**

*Simple statistics*

Regarding reproductives, treatment had no significant effect on the survival of the original queens (Fisher Test: treatment: *N* = 19, *χ^2^* = 1.56, *p =* 0.303) (Figure S1a). Concerning overall queen fecundity, no significant difference between control and protein+ colonies were found regarding the total number of eggs produced within a colony (Mann-Whitney U test: *N* = 20, *W* = 52.50, *p* = 0.879). Egg laying of original queens was not significantly affected by treatment (Mann-Whitney U test: *N* = 19, *W* = 42.00, *p* = 0.805).

For worker fitness, there was no significant effect of treatment on the proportion of workers surviving (Mann-Whitney U test: *N* = 20, *W* = 60.50, *p =* 0.426).

Regarding total fitness, treatment had no significant effect (Mann-Whitney U test: *N* = 20, *W* = 56.50, *p* = 0.623).

Figure S1. Survival of queens. The survival of the original queens was neither significantly affected (a) by treatment nor (b) by stock colony. (a) queens pooled according to treatment; (b) queens sorted according to their genetic background (stock colony) with data from control colonies marked in yellow (circles) and data from protein+ colonies marked in blue (triangles).

*Additional GLMMs for reproductives*

Regarding queens and kings combined, there was no significant effect of treatment, number of workers at the start of the experiment or stock colony on reproductive survival (Binomial GLMM: treatment: *N* = 20, *χ^2^* = 0.77, *p =* 0.381; start: *N* = 20, *χ^2^* = 0.13, *p =* 0.781; stock colony: *N* = 20, *χ^2^* = 1.29, *p =* 0.257).

The analysis of the egg laying rates of original and replacement queens revealed no significant effect of treatment (Figure S2a) (Gaussian LMM: *N* = 22, *χ^2^* = 0.40, *p =* 0.527). The number of workers at the start of the experiment and stock colony also had no significant effects (Gaussian LMM: start: *N* = 22, *χ^2^* = 0.32, *p =* 0.571; stock colony: *N* = 22, *χ^2^* = 0.00, *p =* 1.000). However, Figure S2b indicates that the replacement queens may have had lower egg laying rates than the original queens. Therefore, we decided to consider them separately from the original queens.

Figure S2. Fecundity of original and replacement queens. The egg laying rate of original and replacement queens shown in (a) with violin plots and in (b) sorted according to state of the queen. Control samples are displayed in yellow (circles) and protein+ samples in blue (triangles). Treatment had no significant effect on the combined egg laying rates.

*Effect of genetic background and number of workers on fitness estimates*

Neither the number of workers at the beginning nor the origin of a colony (stock colony) had a significant effect on queen survival (Binomial GLMM: workers: *N* = 19, *χ^2^* = 0.01, *p =* 0.932, stock: *N* = 19, *χ^2^* = 2.66, *p =* 0.103) (Figure S1b).

Stock colony had no significant effect on the total number of eggs produced within a colony (Poisson GLMM: *N* = 20, *χ^2^* = 0.00, *p =* 1.000) (Figure S3a), but the number of workers at the start of the experiment had a significant positive effect (Poisson GLMM: *N* = 20, *χ^2^* = 5.06, *p* = 0.024) (Figure S3b). Egg laying rate of original queens was neither significantly affected by stock colony nor by the number of workers at the start of the experiment (Gaussian LMM: *N* = 19, *χ^2^* = 0.00, *p =* 1.000, start number: *N* = 19, *χ^2^* = 0.44, *p* = 0. 481) (Figure S3c, Figure S3d).

Figure S3. Effects on queen fecundity. Shown are the number of eggs produced within a colony during the whole experimental period (upper panel) and the egg laying rate of original queens standardised by the time a queen was present (lower panel). In (a) and (c) all colonies are sorted according to their genetic background (stock colony) with a jitter to avoid overlapping of points and in (b) and (d) egg production is correlated with the number of workers at the start of the experiment. Control: yellow (circle), protein+: blue (triangle). Stock colony (a) had no significant effect on the total number of eggs produced but (b) the number of workers present at the beginning correlated positively with the number of eggs produced. The individual egg laying rate of original queens was neither affected by (c) stock colony nor (d) starting number of workers.

For the fitness components of the workers, the proportion of surviving workers was not influenced by the genetic background (Gaussian LMM: *N* = 20, *χ^2^* = 0.77, *p =* 0.380) (Figure S4a).

Total colony fitness did not differ between stock colonies (Poisson GLMM: stock: *N* = 20, *χ^2^* = 0.00, *p =* 1.000) (Figure S4b), but the number of workers at the beginning had a significant positive effect (Poisson GLMM: start: *N* = 20, *χ^2^* = 21.86, *p =* 2.94e-06).

Figure S4. Effect of genetic background on survival of workers and total fitness estimates. The data are sorted according to the genetic background of the colonies (stock colony) with a jitter to avoid overlapping of points. Control: yellow, protein+: blue. Colonies with original queens are depicted with circles, colonies with replacement queens with triangles. Stock colony did neither affect (a) the proportion of surviving workers nor (b) the total fitness.

**Feeding results**

*Analysis of both castes combined*

There was no significant correlation, but a tendency that individuals survived better when they fed more on the carcass (Binomial GLMM, treatment: *N* = 10, *χ^2^* = 2.94, *p =* 0.087). The influence of the caste on survival was highly significant (Binomial GLMM, treatment: *N* = 10, *χ^2^* = 11.00, *p =* 9.11e-04) and there might be a stock colony effect (Binomial GLMM, treatment: *N* = 10, *χ^2^* = 3.78, *p =* 0.052).

*Analysis of genetic background effect*

The caste-specific survival analyses revealed that reproductives from some stock colonies survived better than those from others (Binomial GLMM: *N* = 18, *χ^2^* = 16.29, *p =* 5.43e-05) (Figure S5a). However, the egg laying rate of a queen was not significantly affected by stock colony (Gaussian LMM: *N* = 9, *χ^2^* = 0.00, *p =* 1.000) (Figure S5b).

For workers, stock colony had no effect on survival (Binomial GLMM: *N* = 81, *χ^2^* = 0.00, *p =* 1.000) (Figure S5c).

Figure S5**.** Genetic background effect on feeding. The plots show the relationship between the genetic background (stock colony) and (a) relative feeding duration [s] of reproductives, (b) queen fecundity (egg laying rate), and (c) relative feeding duration [s] of workers. We included a jitter to avoid overlapping of data points. Data from surviving individuals are shown in green (diamond shape), data from dead individuals in purple (squares). The genetic background had a significant influence on survival of reproductives but not on individual egg laying rate or workers’ survival.

**Principal component analysis**

The PCA with both tissues combined showed a strong separation between head and fat bodies (PC1: 76%) (see Figure S6a). To test if the tissue effect overshadowed a potential treatment effect, we performed two additional PCAs, one for each tissue. In line with the few DEGs, the PCA with head samples revealed no separation between the two treatments (Figure S6b). A t-test comparing the treatments for both components did not show any significant results (PC1: *t* = -0.75, *p =* 0.467, PC2: *t* = -0.56, *p* = 0.585). The PCA with fat body samples showed a separation between the treatments (PC1: 41*%)* with one control and one protein+ individual not fitting the pattern (Figure S6c). A follow-up t-test comparing the treatments for both components did not show any significant differences (PC1: *t* = 1.53, *p =* 0.191, PC2: *t* = 0.48, *p* = 0.647). In both tissues, we could not see a separation between original and replacement queens.

****Figure S6. PCA. The result of the principal component analysis for (a) both tissues combined, (b) heads and (c) fat bodies. Control queens are displayed in yellow, Protein+ queens are displayed in blue. Stock colonies are indicated with their respective numbers. In (a) Samples from fat bodies are depicted with squares and samples from heads are depicted with diamond shape. There is a clear separation between the tissues along the first component (PC1: 76%) but no separation between treatments. In (b) and (c) the shapes indicate the origin of the queens, whether they were original queens (triangles) or newly developed (circles). There is no separation between treatments in (b) heads and (c) fat bodies. In both tissues, we find a separation between stock colonies, No. 5 in heads, No. 4 in fat bodies.

**Gene expression in heads**

In heads, we only found three DEGs significantly more highly expressed in the treatment group and one DEG in the control group (Supplementary Information: Table S1). There was some evidence for differential expression of genes associated with the TI-J-LiFe network upon protein+ treatment in the head (Figure S7).

Figure S7. Heatmap for differential gene expression analysis in heads. Shown are the results of the differential gene expression analysis for genes related to IIS, TOR and genes indicating high JH titres and fecundity in C. secundus. The colour symbolizes the log2foldchange value with red indicating an upregulation and blue a downregulation of the respective gene under protein+ conditions. The adjusted p-values are given in the tiles. None of these genes was significantly differentially expressed
